# DNA-Free Genome Editing of *Brassica oleracea* and *B. rapa* Protoplasts Using CRISPR-Cas9 Ribonucleoprotein Complexes

**DOI:** 10.3389/fpls.2018.01594

**Published:** 2018-11-05

**Authors:** Jana Murovec, Katja Guček, Borut Bohanec, Monika Avbelj, Roman Jerala

**Affiliations:** ^1^Department of Agronomy, Biotechnical Faculty, University of Ljubljana, Ljubljana, Slovenia; ^2^Department of Synthetic Biology and Immunology, National Institute of Chemistry, Ljubljana, Slovenia

**Keywords:** RGEN, CRISPR/Cas9, genome editing, NGS, ribonucleoproteins, protoplast, genus *Brassica*, *B. napus*

## Abstract

The CRISPR/Cas9 genome editing system has already proved its efficiency, versatility and simplicity in numerous applications in human, animal, microbe and plant cells. Together with the vast amount of genome and transcriptome databases available, it represents an enormous potential for plant breeding and research. Although most changes produced with CRISPR/Cas9 do not differ from naturally occurring mutations, the use of transgenesis during varietal development can still trigger GMO legislation in countries that rely on process-based regulation. Moreover, stable integration of DNA coding for genome-editing tools into plant genomes can result in insertional mutagenesis, while its prolonged expression can cause mutations in off-target sites. These pitfalls can be avoided with the delivery of preassembled ribonucleoprotein complexes (RNPs) composed of purified recombinant enzyme Cas9 and *in vitro*-transcribed or synthesized sgRNA. We therefore aimed to develop a DNA-free protocol for site-directed mutagenesis of three species of the genus *Brassica* (*B. oleracea, B. napus*, and *B. rapa*) with the use of RNPs. We chose cabbage, rapeseed and Chinese cabbage as species representatives and introduced RNPs into their protoplasts with PEG 4000. Four sgRNAs targeting two endogenous genes (the *FRI* and *PDS* genes, two sgRNAs per gene) were introduced into all three species. No mutations were detected after transfection of rapeseed protoplasts, while we obtained mutation frequencies of 0.09 to 2.25% and 1.15 to 24.51% in cabbage and Chinese cabbage, respectively. In both species, a positive correlation was displayed between the amount (7.5, 15, 30, and 60 μg) of Cas9 enzyme and sgRNA introduced and mutation frequency. Nucleotide changes (insertions and deletions) were detected 24 h after transfection and did not differ 72 h after transfection. They were species-, gene- and locus-dependent. In summary, we demonstrated the suitability of RNP transfection into *B. oleracea* and *B. rapa* protoplasts for high-efficiency indel induction of two endogenous genes. Due to the relatively high mutation frequencies detected (up to 24.51%), this study paves the way for regeneration of precisely mutated *Brassica* plants without the use of transgenesis.

## Introduction

CRISPR/Cas9-mediated genome editing is revolutionizing life sciences by providing new, precise, facile and high-throughput tools for genetic modification of genomes of numerous species from different kingdoms. This novel gene-editing tool belongs to the type II bacterial adaptive immune system and consists of a Cas9 enzyme, representing the scissors, and an RNA complex as the precise targeting component. tracrRNA and crRNA, RNA complexes, guide endonuclease Cas9 to target the desired genome site, which bears a short PAM sequence that is recognized by the Cas9 protein. Its action relies on the site-specific introduction of double-stranded DNA breaks and subsequent repair of disrupted genome integrity by error-prone non-homologous end-joining (NHEJ) or homology-directed repair (HDR) (Hsu et al., 2013). Due to guide RNA (sgRNA, the fusion product of tracrRNA and crRNA) being used as the targeting method, CRISPR can also be used for multiplexing, targeting several DNA regions simultaneously.

So far, the most broadly used methods for employing CRISPR/Cas9 as a genome-editing tool in plants have relied on the stable transformation of DNA expression vectors with standard transformation methods, such as *Agrobacterium tumefaciens*, biolistics and PEG-mediated transformation of protoplasts. These techniques present specific concerns or have limitations in their use. Stable transformation of plants is a lengthy process that results in regeneration of transgenic plants with expression cassettes integrated into their genomes at one or more unpredictable sites. It can cause insertional mutagenesis and unwanted modifications of off-target sequences due to prolonged expression of CRISPR/Cas9 constructs. Although for most genome-editing applications, their constant presence in the genomes is not needed, and the DNA expression cassettes can be bred out in later stages, it additionally prolongs the whole procedure and is not possible in self-incompatible, dioecious or asexually reproducing plant species (such as grape, potato, fruit trees and others).

Moreover, while the presence of targeted mutations in final products does not present an obstacle for the release of such improved varieties, the integration of genome-editing vectors into plant genomes during their development is unwanted. Such plants fall within the scope of the GMO legislation in countries that rely on process-based regulation of GMOs (Wolt et al., 2016). It is still a topic of debate but, for now, it limits the usefulness of genome editing in plant breeding, agriculture and horticulture.

As an alternative solution, a plant DNA-free genome-editing technique was introduced by Woo et al. (2015). They induced stable nucleotide changes in *Arabidopsis thaliana*, tobacco, lettuce and rice by integration of preassembled Cas9 enzyme from *Streptococcus pyogenes* with *in vitro*-transcribed sgRNA (the ribonucleoprotein complex, RNP) (Woo et al., 2015). It was followed by reports about successful mutagenesis with RNPs in grapevine and apple (Malnoy et al., 2016), maize (Svitashev et al., 2016), *Petunia* × *hybrida* (Subburaj et al., 2016), wheat (Liang et al., 2017), and potato (Andersson et al., 2018). In 2016, the use of RNPs with Cpf1 enzyme was reported for soybean and wild tobacco (Kim et al., 2017). In the published research, the authors have used PEG-mediated transfection of protoplasts (Woo et al., 2015; Malnoy et al., 2016; Subburaj et al., 2016; Kim et al., 2017; Andersson et al., 2018) or biolistics (Svitashev et al., 2016; Liang et al., 2017) to introduce RNPs, and some of them have succeeded in regenerating mutated plants from treated cells.

The genus *Brassica* comprises a large number of species and subspecies that are consumed either as shoots, leaves, roots or turnip roots or in the form of seeds. Vegetative plant parts are merchandized mainly as raw products, whereas generative parts are marketed predominantly in a processed form as oil, meal, powder, protein or condiment (Möellers, 2017). They are widely used as food and fodder, and their contribution to human nutrition and their health benefits are highly appreciated (Kumar and Andy, 2012). The species have diversified into a large number of agriculturally important morphotypes due to domestication and further breeding. The primary vegetable species among brassicas is the species *Brassica oleracea*, which includes morphotypes of cabbage, kale, Chinese kale, savoy cabbage, Brussels sprouts, kohlrabi, broccoli, and cauliflower. The species *B. rapa* comprises morphotypes of Chinese cabbage, pak choi, turnip, and oilseeds. The species *B. napus* is an allopolyploid that was formed ∼7,500 years ago by hybridization between *B. oleracea* and *B. rapa* (Chalhoub et al., 2014). It also includes several morphotypes (rapeseed, rutabaga, and fodder rape), with rapeseed (canola) being the economically most important as the oil crop with the third highest production quantity (FAOSTAT, 2014).

Despite their high economic importance, modern biotechnological approaches for breeding and research of *Brassica* species are still lacking. The few studies published on genome editing relied on stable transformation with *A. tumefaciens* (Sun et al., 2013; Lawrenson et al., 2015; Braatz et al., 2017; Kirchner et al., 2017; Yang et al., 2017).

Our study aimed to develop a protocol for DNA-free genome editing of different species of the genus *Brassica*. Due to their high economic value, we chose cabbage and Chinese cabbage as representatives of the species *B. oleracea* and *B. rapa*, respectively, and rapeseed as representative of *B. napus*. We targeted two genes, the phytoene desaturase gene (*PDS*) involved in the carotenoid biosynthesis pathway, and the vernalization determinant FRIGIDA (*FRI*) gene, each at different loci. To develop a standard protocol for different *Brassica* species, we designed sgRNAs and primers complementary to coding regions that are preserved among the three species studied.

## Materials and Methods

### Expression of Cas9 Protein

The pET28b-His-Cas9 vector containing recombinant Cas9 gene was obtained from Addgene (plasmid ID 47327) and expressed in *E. coli* Rosetta cells (Novagen). His-tagged Cas9 protein was expressed using the auto-induction medium ZYP-5052 (Studier, 2005). His-tagged Cas9 protein was purified using Ni-NTA agarose beads and dialyzed in dialysis buffer (20 mM Tris pH 8, 200 mM KCl, 10 mM MgCl_2_) (Gagnon et al., 2014). The enzyme was quantified with a Bradford assay.

### Target Site Selection and *in vitro* Transcription of sgRNA

*FRI* gene, accession Bra035723 (Sun et al., 2013), was obtained from the *Brassica* database^1^. It was aligned with other *Brassica* sequences deposited in the NCBI GenBank with CLC Genomics software (Qiagen) to design ‘FRI-Seq’ sequencing primers (Table 1). ‘PDS-Seq-Dig’ sequencing primers (Table 1) were designed by aligning *B. napus PDS* gene (HM989807) with other GenBank sequences. PCR products were cloned in a pGEM-T-Easy Vector System (Promega), and the plasmid DNA was Sanger-sequenced.

**Table 1:** Sequences of primers used for amplification of CRISPR target loci of *FRI* and *PDS* genes of *B. oleracea, B. napus*, and *B. rapa*.

Target gene	Sequence (5′–3′)	Expected product size
FRI-Seq	For GTGCCTACAAACACGGAAAT	∼1,200 bp
	Rev AAGGGACATGCAAATGCTAT	
FRI-Dig-BO	For AAACGCCACTACGACGACTT	520 bp
	Rev CCTCGGCTTCATCCTTGATA	
FRI-Dig-BR	For AAACGCCACTACGACGACTT	496 bp
	Rev CCTCGGCTTGATCCTTGATA	
FRI-NGS	For AACGATGCTTCCGGAGAAA	194 bp (BR)
	Rev TCCTTGGCTAGCTTCAGAGC	212 bp (BO)
PDS-Seq-Dig-BO	For CCGAGAGCCAGAAAACACA	977 bp
	Rev GAATTGCACGCGTAGAGTGA	
PDS-Seq-Dig-BR	For ATCCTCATCCTTCCATGCAG	1075 bp
	Rev CTCCATTTTGGGATTGGCTA	
PDS-NGS	For CAGATTCCTTGAAGCAGTT	218 bp
	Rev TTTTGAATGAAACAGACAGAGACC	


The sequences of *FRI* and *PDS* genes obtained from three *Brassica* species (*B. oleracea, B. napus*, and *B. rapa*) were aligned with CLC Genomics software, and potential target sites in conserved regions of *FRI* and *PDS* genes were designed with CLC Genomics Workbench and CRISPR RGEN Tools Cas-Designer (Park et al., 2015).

Double-stranded template DNA for *in vitro* transcription was obtained by annealing two overlapping oligonucleotides as described by Gagnon et al. (2014). sgRNAs (Table 2) were transcribed *in vitro* with a HiScribe^TM^ T7 Quick High Yield RNA Synthesis Kit (NEB), purified with a MEGAclear^TM^ Kit (Ambion) according to the manufacturer’s instructions and quantified using a NanoVue Plus spectrophotometer (GE Healthcare).

**Table 2:** List of sgRNAs designed to target *FRI* and *PDS* genes of *B. oleracea, B. napus*, and *B. rapa*.

			Expected size of cleaved PCR products (bp)	

Target gene	sgRNA name	Target sequence (5′–3′) with PAM (in bold)	*B. oleracea*	*B. rapa*
*FRI*	FRI1^∗^	TGCGAGTTGATGTGCAGCAA**AGG**	278, 242	272, 224
*FRI*	FRI2	CTCCTTTGGCGGCGATTGTG**TGG**	357, 163	342, 154
*FRI*	FRI3	CGATCGGGAGGAGGGAGACT**CGG**	429, 91	405, 91
*FRI*	FRI4^∗^	GCTCTTCAATCAGCTTAGCT**CGG**	287, 233	269, 227
*FRI*	FRI5	GAAGCGAAACCTGCCTCGCA**GGG**	419, 101	401, 95
*PDS*	PDS1.1^∗^	TGTGTTTGGGAATGTTTCCG**CGG**	759, 218	799, 276
*PDS*	PDS1.2^∗^	GAGGAGTGCTGGTCCTTTGC**AGG**	621, 356	661, 414


^∗^Selected sgRNAs used for transfection of protoplasts.

### *In vitro* Digestion Assay

An *in vitro* digestion assay was performed to assess *in vitro* cleavage activity of the purified Cas9 enzyme and *in vitro*-synthesized sgRNAs. Target sites for *FRI* and *PDS* genes were amplified with the primers ‘FRI-Dig-BO,’ ‘FRI-Dig-BR,’ ‘PDS-Seq-Dig-BO,’ and ‘PDS-Seq-Dig-BR’ listed in Table 1 and column-purified (Illustra GFP PCR DNA and gel band purification kit, GE Healthcare); 100 ng of purified PCR products was incubated with 160 ng or 1 μg Cas9 and 160 ng or 1 μg sgRNA in 1× NEBuffer 3 with BSA for 1 h at 37°C. It was followed by enzyme deactivation at 65°C for 10 min and visualization of bands with 2% agarose gel electrophoresis.

### Protoplast Isolation and Transfection

Cabbage (*B. oleracea* var. *capitata* f. *alba*) ‘Varaždinsko,’ Chinese cabbage (*Brassica rapa* subsp. *pekinensis*) and oilseed rape (*Brassica napus*) ‘Topaz’ plants were grown *in vitro* on hormone-free Murashige and Skoog medium with 30 g/l sucrose and 8 g/l agar, pH 5.8, at 20°C and with a 16-h photoperiod. Young, fully developed leaves were chopped and immersed in cell-wall digestion enzyme solution composed of 0.5% Cellulase Onozuka RS (Yakult Pharmaceuticals), 0.1% Pectolyase Y-23 (Duchefa), 2 mM MES (pH 5.7), 3 mM CaCl_2_ and 0.4 M mannitol. Vacuum infiltration for 30 min was followed by incubation for 2.5 h on a rotary shaker at 30 rpm and 25°C. After filtration through a 40-μm nylon mesh, the suspension was centrifuged for 5 min at 130 × *g*; the pellet was resuspended in 0.5 M sucrose with 1 mM MES (pH 5.7) and covered with W5 solution (2 mM MES pH 5.7, 154 mM NaCl, 125 mM CaCl_2_, 5 mM KCl). After centrifugation at 190 × *g* for 5 min, the protoplasts were collected from the interface layer, then resuspended in W5 solution and centrifuged at 130 × *g* for 5 min. The harvested protoplasts were resuspended in MMG solution (4 mM MES pH 5.7, 0.4 M mannitol, 15 mM MgCl_2_) and incubated on ice for the duration of the transfection experiments. The viability of protoplasts was determined with FDA staining (final concentration 1 μg/ml), and the concentration of protoplasts in suspension was counted using a hemocytometer.

For each transfection experiment, 5 × 10^5^ protoplasts in 200 μl of MMG solution were used. To prepare the RNP complexes, the purified Cas9 protein (0 to 60 μg) was mixed with *in vitro*-transcribed sgRNA (0 to 60 μg) in NEBuffer 3 and incubated for 15 min at 25°C. They were mixed with protoplast suspensions before addition of an equal volume of 40% PEG 4000, then mixed gently and incubated at room temperature in the dark for 15 min. An equal volume of W5 solution was added twice, then mixed and centrifuged at 80 × *g* for 5 min. The supernatant was discarded; the protoplasts were rinsed with CPP medium (Kiełkowska and Adamus, 2012) and finally incubated in CPP solution in the dark at 25°C for 24 or 72 h.

### Targeted Deep Sequencing and Calculation of Mutation Efficiency

DNA was isolated from protoplasts 24 or 72 h after transfection, with a Qiagen DNeasy Plant Mini Kit. CRISPR target sites (∼200 bp) were amplified with Q5^®^ High-Fidelity DNA Polymerase (NEB) and the primers FRI-NGS-For (5′-AACGATGCTTCCGGAGAAA-3′) and FRI-NGS-Rev (5′-TCCTTGGCTAGCTTCAGAGC-3′), and PDS-NGS-For (5′-CAGATTCCTTGAAGCAGTT-3′) and PDS-NGS-Rev (5′-TTTTGAATGAAACAGACAGAGACC-3′) for *FRI* and *PDS* genes, respectively. Amplified PCR products were sequenced using the Illumina HiSeq platform at GATC Biotech (Konstanz, Germany). Mutations induced at the protospacer sites were analyzed with CRISPR RGEN Tools Cas-Analyzer software (Park et al., 2017) and CRISPResso (Pinello et al., 2016). Three biological replications were performed, and the percentages of mutations were presented as average values of indels around the CRISPR RNP cleavage sites.

## Results

Sequencing of *B. oleracea, B. napus*, and *B. rapa* revealed numerous sequence differences in the coding regions of *FRI* and *PDS* genes, thus restraining the number of common target sites for sgRNAs and for targeted deep sequencing primers. As shown in Supplementary Figure 1A, the universal primers FRI-NGS amplified one 212 bp-long *FRI* allele in *B. oleracea*, one 194 bp-long allele in *B. rapa* and two alleles in *B. napus*. *B. napus* contained the same 212 bp-long allele as *B. oleracea* (with 100% nucleotide identity) while the second allele was almost identical to the *B. rapa* allele, but with an 18 bp-long deletion. The sequences of *PDS* gene also showed polymorphism between the species, with *B. napus* containing alleles from each parental species, as shown in Supplementary Figure 1B. Amplification with PDS-NGS primers produced two alleles, both 218 bp long, with five SNPs between *B. oleracea* and *B. rapa* alleles. One G/C SNP was also observed within *B. rapa* sequences, as shown in Supplementary Figure 1B.

**FIGURE 1:**
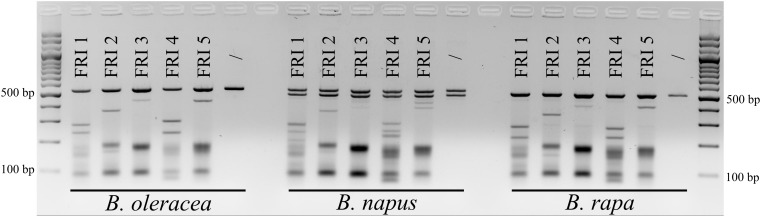
Results of *in vitro* digestion assay. The gene *FRI* was PCR-amplified from cabbage (*B. oleracea*), oilseed rape (*B. napus*), and Chinese cabbage (*B. rapa*) DNA and treated with RNPs preassembled with five different sgRNAs (FRI1 to FRI 5). For each species, non-treated samples were used as negative controls (/).

Based on aligned sequences of the three species, seven different sgRNAs (Table 2) were designed and synthesized for targeting the first exons of the *FRI* gene (five sgRNAs) and of the *PDS* gene (two sgRNAs). Cleavage activity of sgRNAs FRI1 to FRI5 was tested with the *in vitro* digestion assay using 160 ng of sgRNA and 160 ng of Cas9 enzyme. The results obtained are presented in Figure 1. They show that all the sgRNAs tested were able to cleave PCR products of the *FRI* gene obtained from all three species included in our study and that individual sgRNAs cleave PCR products from different species with the same cleavage efficiency. Different sgRNAs differed in their cleavage efficiency; sgRNA-FRI1 and sgRNA-FRI4 showed the highest activity and were therefore chosen for subsequent experiments on transfection of protoplasts of *B. oleracea, B. napus*, and *B. rapa*.

Due to incomplete digestion of PCR products, the following *in vitro* digestion assays with sgRNA-FRI1, sgRNA-FRI4, sgRNA-PDS1, and sgRNA-PDS2 were performed with a higher concentration of Cas9 (1 μg) and sgRNA (1 μg), and the digested products were column-purified before loading on 2% agarose gel. PCR products for digestion of the *FRI* gene were amplified with the primers ‘FRI-Dig-BO’ and FRI-Dig-BR’ (Table 1), while PCR products for digestion of the *PDS* gene were amplified using the same primers as were used for sequencing (‘PDS-Seq-Dig-BO’ and ‘PDS-Seq-Dig-BR,’ Table 1). The results showed that all four sgRNAs tested were able to cleave PCR products from all three species and that the cleavage was complete when using a higher amount of sgRNA and Cas9 (Supplementary Figure 2).

Since sgRNA-FRI1, sgRNA-FRI4, sgRNA-PDS1, and sgRNA-PDS2 showed high cleavage activity *in vitro*, they were used for genome editing of *B. oleracea, B. napus*, and *B. rapa* protoplasts. They were transfected into the isolated protoplasts with PEG 4000, and the results were displayed as the percentage of indels detected at the cleavage site based on targeted deep sequencing and analysis with Cas-Analyzer.

**FIGURE 2:**
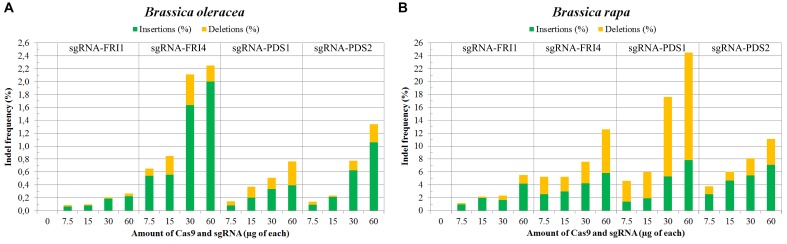
Mutation frequencies in *B. oleracea* **(A)** and in *B. rapa* **(B)** measured by targeted deep sequencing and Cas-Analyzer. Percentages of induced insertions and deletions are represented in green and yellow, respectively.

In *B. napus* protoplasts, no indels were detected for any of the sgRNAs used at any concentration, while in *B. oleracea* and *B. rapa*, the indel frequencies varied between 0.09 and 24.51%. Sequences from control samples – protoplasts transfected with sterile water – did not show mutations at target sites, and the calculated indel frequencies for all control samples were 0.00%. Substantially higher mutation frequencies were detected in *B. rapa* (1.15 to 24.51%) as compared to *B. oleracea* (0.09 to 2.25%) for both genes, all sgRNAs and all concentrations used in our experiments. Within both species, mutation frequencies were gene- and locus-dependent and correlated with the amount of RNPs used. Detailed results obtained with three biological replicates for each combination of species, sgRNA and concentration used are presented in Table 3 and Figures 2A,B. They show that RNPs can induce mutations in protoplasts of *B. oleracea* and *B. rapa* and that with the protocol we used, higher indel frequencies can be obtained in *B. rapa* as compared to *B. oleracea*. For all sgRNAs used, the frequency of indels positively correlated with the amount of RNPs transfected. The most efficient sgRNA was sgRNA-PDS1, with indel percentages up to 24.51% in *B. rapa*. Among the two sgRNAs used to target the *FRI* gene, sgRNA-FRI-4 was more efficient in both species.

**Table 3:** Mutation rates based on deep sequencing of target regions in *FRI* and *PDS* genes and analysis with Cas-Analyzer.

Species	Target gene	sgRNA	Amount of sgRNA and Cas9 enzyme (μg)	No. of reads analyzed	No. of insertions	No. of deletions	Indel frequency (%)
*B. oleracea*	*FRI*	FRI1	0	119,218	0	0	0.00
Cabbage		FRI1	7.5	253,135	164	53	0.09
		FRI1	15	260,476	205	42	0.09
		FRI1	30	111,095	207	24	0.21
		FRI1	60	126,052	276	59	0.27
		
		FRI4	0	119,158	0	1	0.00
		FRI4	7.5	92,061	497	104	0.65
		FRI4	15	177,088	987	512	0.85
		FRI4	30	163,280	2,674	775	2.11
		FRI4	60	102,222	2,044	257	2.25

*B. oleracea*	*PDS*	PDS1	0	163,019	0	0	0.00

Cabbage		PDS1	7.5	214,791	173	138	0.14
		PDS1	15	203,212	413	341	0.37
		PDS1	30	219,727	732	381	0.51
		PDS1	60	134,761	526	505	0.77
		
		PDS2	0	155,112	0	0	0.00
		PDS2	7.5	129,478	119	57	0.14
		PDS2	15	157,714	335	33	0.23
		PDS2	30	107,439	674	157	0.77
		PDS2	60	142,224	1,496	395	1.33

*B. rapa*	*FRI*	FRI1	0	162,370	3	1	0.00

Chinese		FRI1	7.5	238,999	2,243	505	1.15
cabbage		FRI1	15	250,542	4,954	465	2.16
		FRI1	30	323,059	5,299	2,269	2.34
		FRI1	60	196,782	8,258	2,540	5.49
		
		FRI4	0	162,313	1	0	0.00
		FRI4	7.5	145,754	3,704	3,904	5.22
		FRI4	15	227,571	6,718	5,164	5.22
		FRI4	30	202,560	8,580	6,708	7.55
		FRI4	60	244,590	14,281	16,483	12.58

*B. rapa*	*PDS*	PDS1	0	100,126	0	0	0.00

Chinese		PDS1	7.5	407,818	5,645	13,067	4.59
cabbage		PDS1	15	367,804	7,056	14,867	5.96
		PDS1	30	241,779	12,749	29,890	17.64
		PDS1	60	169,020	13,280	28,148	24.51
		
		PDS2	0	94,671	0	0	0.00
		PDS2	7.5	101,830	2,606	1,241	3.78
		PDS2	15	199,875	9,370	2,723	6.05
		PDS2	30	167,078	9,156	4,402	8.11
		PDS2	60	146,015	10,343	5,830	11.08


The results are the summary of three biological replicates.

**FIGURE 3:**
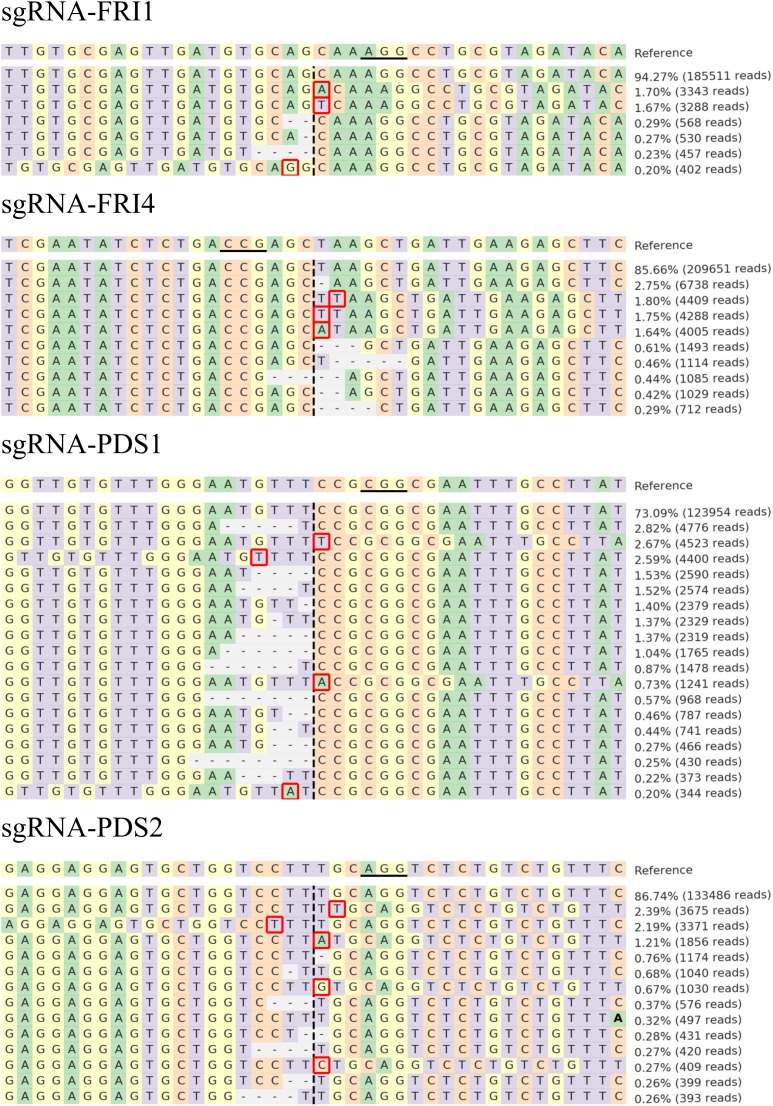
Distribution of the most frequent alleles identified with CRISPResso around cleavage sites in *B. rapa.* Sequences were obtained after transfection with 60 μg Cas9 preassembled with 60 μg of sgRNA-FRI1, sgRNA-FRI4, sgRNA-PDS1, or sgRNA-PDS2. The horizontal line indicates the PAM region, the vertical dashed line indicates the predicted cleavage site. Mutations are shown in bold font (substitutions), highlighted with red rectangles (insertions), or marked with horizontal dashed lines (deletions).

The sequences were further analyzed with CRISPResso, and comparable results were obtained. Figure 3 presents the distribution of the most frequent alleles found around the cleavage site in *B. rapa* after transfection with the highest concentration of RNPs used (60 μg of Cas9 preassembled with 60 μg of sgRNA). Most of the insertions detected in the 40-bp region surrounding the cleavage sites were 1-bp long and were located one to four nucleotides downstream or upstream of the cleavage site. The deletions detected were longer (1–8 bp) than the insertions (1 bp) and started at the cleavage site or one base apart from it.

To evaluate if the time for which protoplasts were cultured after the introduction of RNPs affects mutation frequencies, we transfected 15 μg of sgRNA-PDS2 and 15 μg of Cas9 into *B. rapa* protoplasts. DNA was isolated after 24 h and after 72 h; for each time-course, three biological replicates were performed as in the experiments presented above. The average mutation frequency in DNA isolated 24 h after transfection was 4.89% (1. replicate 3.16%; 2. replicate 4.61%; and 3. replicate 6.91%), while 72 h after transfection, the average indel frequency was 4.30% (1. replicate 2.55%; 2. replicate 4.28%; and 3. replicate 6.07%) (Supplementary Figure 3) which was not significantly different (*p* = 0.711).

## Discussion

Our experiments demonstrated the suitability of the autoinduction protocol for purification of recombinant Cas9 enzyme in *E. coli* and of *in vitro* transcription for the production of sgRNAs. We showed that with the protocols used, high-quality RNP components can be obtained at moderate cost. Previous experiments on the transfection of RNPs into plants relied on commercially available Cas9 enzyme (Woo et al., 2015; Malnoy et al., 2016; Subburaj et al., 2016; Svitashev et al., 2016; Andersson et al., 2018).

Seven sgRNAs were designed for targeting two endogenous *Brassica* genes (*FRI* and *PDS*), and four of them (two per gene) were transfected into protoplasts of *B. oleracea, B. napus*, and *B. rapa*. The preassembled RNPs were able to cleave PCR products completely and induced *in vivo* mutations rates of 0.09 to 24.51% in *B. oleracea* and *B. rapa*. To the best of our knowledge, this is the first report about direct delivery of the CRISPR/Cas9 system as RNPs into any species of the genus *Brassica*, and the first demonstration of site-directed mutagenesis of endogenous *Brassica* genes using RNPs.

Four sgRNAs (sgRNA-FRI1, sgRNA-FRI4, sgRNA-PDS1, and sgRNA-PDS2) and four different amounts of sgRNA and Cas9 enzyme were tested (7.5, 15, 30, and 60 μg), and they stimulated the occurrence of insertions and deletions at target sites (Table 3). Most mutations caused 1 to 8 base pair-long frame shifts (Figure 3) that would disrupt the reading frame of mRNA and cause loss of function in alleles.

In this report, we are the first to show a positive correlation between the amount of RNPs used and the efficiency of site-directed mutagenesis in plants, as ascertained with targeted deep sequencing. In previous reports referring to the use of RNPs in plant protoplasts, the authors used one or more different concentrations of RNPs, but the effect pattern on mutation frequencies was not clearly demonstrated. In *Arabidopsis*, 71% indels were obtained with 20 μg Cas9, and 54% indels with 60 μg Cas9 24 h after transfection. At a later time point (72 h after transfection), the percentages of indels recorded with the two different amounts of RNPs were almost equal: 69 and 71% indel frequencies by introducing 20 and 60 μg Cas9, respectively. The authors prepared RNPs by mixing Cas9 protein (10–60 μg) with double the mass amount of sgRNA (20–120 μg) (Woo et al., 2015). In *Petunia* × *hybrida*, the RNPs used were composed of 90 μg Cas9 and 50 μg sgRNA and produced from 2.4 to 21% mutation rates as detected with T7EI. When analyzing the same DNA samples with targeted deep sequencing, mutation rates of 5.3 to 17.83% were obtained. Only one concentration of RNPs was tested, but locus (sgRNA) dependency was observed (Subburaj et al., 2016). In grape and apple protoplasts, three different concentrations of Cas9 and sgRNA were used: 1:1 (30 μg Cas9 + 30 μg sgRNA), 1:3 (30 μg Cas9 + 90 μg sgRNA) and 3:1 (90 μg Cas9 + 30 μg sgRNA). No conclusions about the best ratio of Cas9:sgRNA could be drawn from the results as they differed substantially between loci. In the other reports about the use of RNPs (Cas9 + sgRNA) in plants, biolistics was used (Svitashev et al., 2016; Liang et al., 2017) so their results are therefore incomparable to our results about transfection of protoplasts with PEG.

In our experiments, the activity of the same RNP when transfected into protoplasts of different species, differed substantially. It correlated with the viability of protoplasts after isolation and transfection. The lowest viability (50% and lower) of protoplasts was observed in *B. napus*, and the results of targeted deep sequencing did not confirm any mutations. Using the same protoplast isolation protocol, we obtained higher protoplasts yield and viability from *B. rapa* leaves as compared to *B. oleracea* leaves and in both species, the viability dropped after transfection with RNPs (data not shown). The results exposed the significance of good protoplast preparation and handling.

Time-course analysis in *B. rapa* showed similar mutation frequencies after 24 h (4.89%) and after 72 h (4.30%) of culture, which confirmed the results obtained in *A. thaliana* (Woo et al., 2015). The authors suggested that RNPs induce mutations before a full cycle of cell division is completed (Woo et al., 2015); studies on human cells showed robust editing within 3 h after transfection of RNPs and a plateau by 24 h, while plasmid-mediated editing persisted in cells for at least 72 h (Kim et al., 2014).

This is the first report on site-directed mutagenesis using RNPs in cabbage (*B. oleracea* L.) and Chinese cabbage (*B. rapa* L.) in two endogenous reporter genes: the phytoene desaturase gene (*PDS*) and the vernalization determinant FRIGIDA (*FRI*) gene. Since relatively high mutation frequencies were obtained, the procedure can be further used for targeting genes of agronomic interest followed by regeneration of edited protoplasts. Efficient protoplast regeneration protocols exists for *Brassica* species (Glimelius, 1999; Sheng et al., 2011; Lian et al., 2012; Kiełkowska and Adamus, 2017). Until now they have been used in somatic hybridization experiments aimed at overcoming barriers in sexual crosses or to modify cytoplasmic traits by altering organelle populations (Lian et al., 2015; Kang et al., 2017) and in experiments of genetic transformation by direct DNA uptake (Nugent et al., 2006). Combined with our protocol for DNA-free genome editing of *Brassica*, they will enable the development of plants with edited phenotypes without the use of transgenesis.

## Conclusion

We have shown that RNPs can be used for targeted CRISPR/Cas9 genome editing of two endogenous genes (*FRI* and *PDS*) in cabbage and Chinese cabbage protoplasts. Local insertion and deletion mutations (indels) were induced even with the lowest amount of Cas9 and sgRNA used (7.5 μg each), and a positive correlation between concentration (7.5 to 60 μg each) and indel frequency was observed. By targeting preserved coding regions, we were able to use the same sgRNAs and primers for different *Brassica* species (*B. oleracea, B. napus*, and *B. rapa*). However, although *in vitro* digestion assays on PCR products showed comparable cleavage efficiencies for all treated species, *in vivo* mutation frequencies differed among species, depending on the viability of protoplasts after isolation. Mutations were detected 24 h after transfection of RNPs into protoplasts, and their frequency did not change significantly 72 h after transfection. Further studies will be focused on site-directed mutagenesis of trait-specific genes and on the regeneration of edited protoplasts.

## Author Contributions

JM conceived the research and wrote the manuscript with the cooperation and support of all co-authors. JM, KG, and MA designed and performed the experiments. BB and RJ provided materials and funding and supervised the research. All authors read and approved the manuscript.

## Conflict of Interest Statement

The authors declare that the research was conducted in the absence of any commercial or financial relationships that could be construed as a potential conflict of interest.

## Footnotes

1 http://brassicadb.org/
