# Supplementary material for: DNA-Free Genome Editing of Brassica oleracea and B. rapa Protoplasts Using CRISPR-Cas9 Ribonucleoprotein Complexes
Source: Front Plant Sci. 2018 Nov 5;9:1594. doi: 10.3389/fpls.2018.01594 (PMC6230560; doi:10.3389/fpls.2018.01594)
Supplement: Supplementary file 1 [file Data_Sheet_1.PDF]

DNA-Free Genome Editing of *Brassica oleracea* and *Brassica rapa* Protoplasts Using CRISPR-Cas9 Ribonucleoprotein Complexes

Jana Murovec, Katja Guček, Borut Bohanec, Monika Avbelj, Roman Jerala

SUPPLEMENTARY MATERIAL

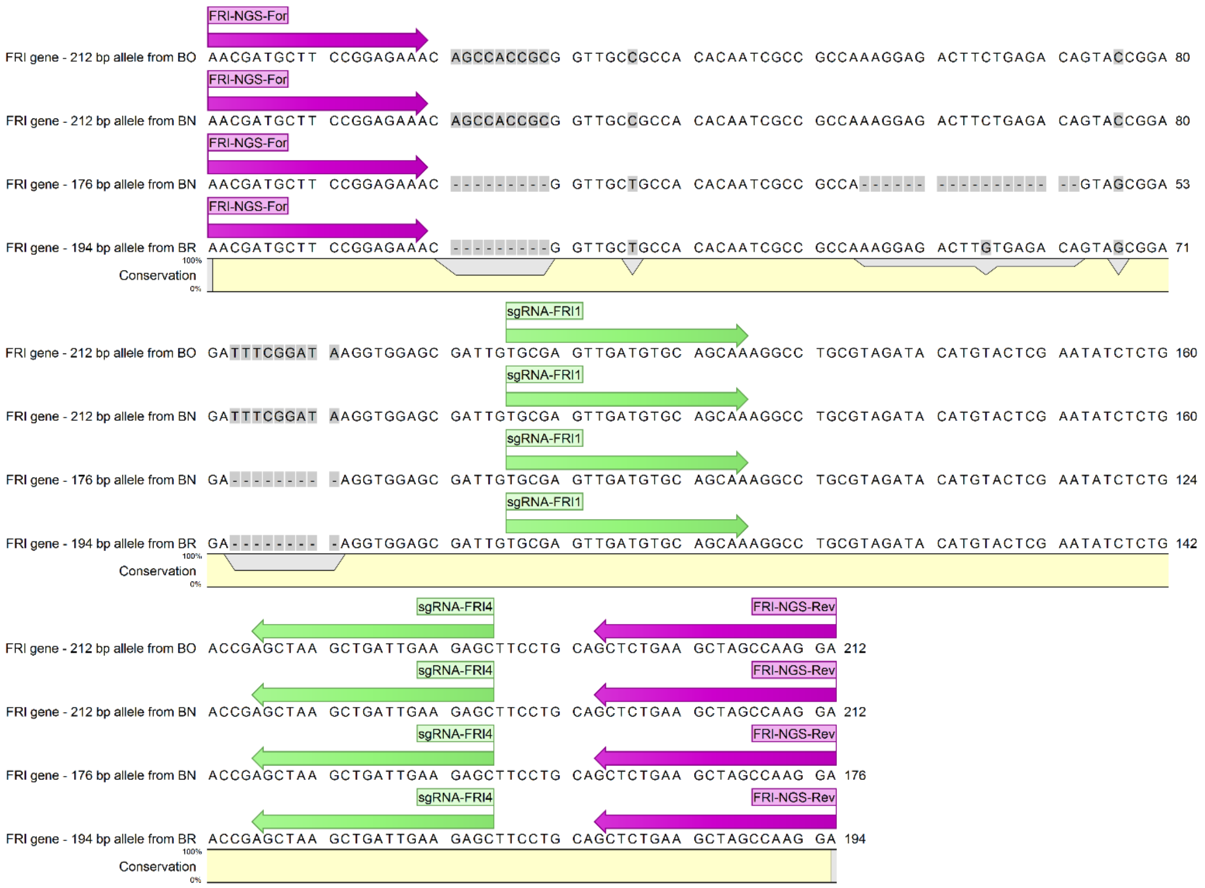

Supplementary Figure 1A Alignment of *FRI* gene sequences flanking sgRNA-FRI1 and sgRNA-FRI4 target sites. Primers FRI-NGS are in pink, sgRNAs are in green and non-identical nucleotides in grey.

DNA-Free Genome Editing of *Brassica oleracea* and *Brassica rapa* Protoplasts Using CRISPR-Cas9 Ribonucleoprotein Complexes

Jana Murovec, Katja Guček, Borut Bohanec, Monika Avbelj, Roman Jerala

SUPPLEMENTARY MATERIAL

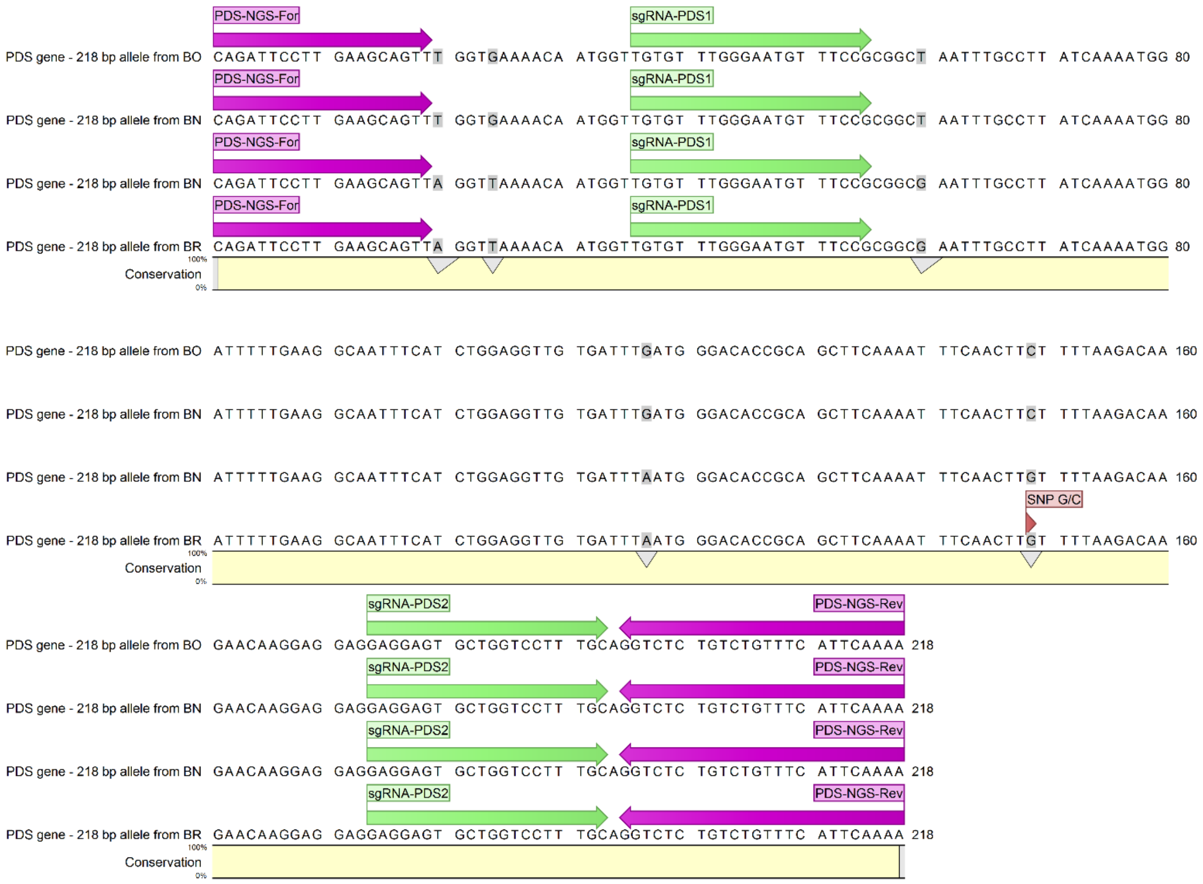

Supplementary Figure 1B Alignment of *PDS* gene sequences flanking sgRNA-PDS1.1 and sgRNA-PDS1.2 target sites. Primers PDS-NGS are in pink, sgRNAs are in green and non-identical nucleotides in grey.

DNA-Free Genome Editing of *Brassica oleracea* and *Brassica rapa* Protoplasts Using CRISPR-Cas9 Ribonucleoprotein Complexes

Jana Murovec, Katja Guček, Borut Bohanec, Monika Avbelj, Roman Jerala

SUPPLEMENTARY MATERIAL

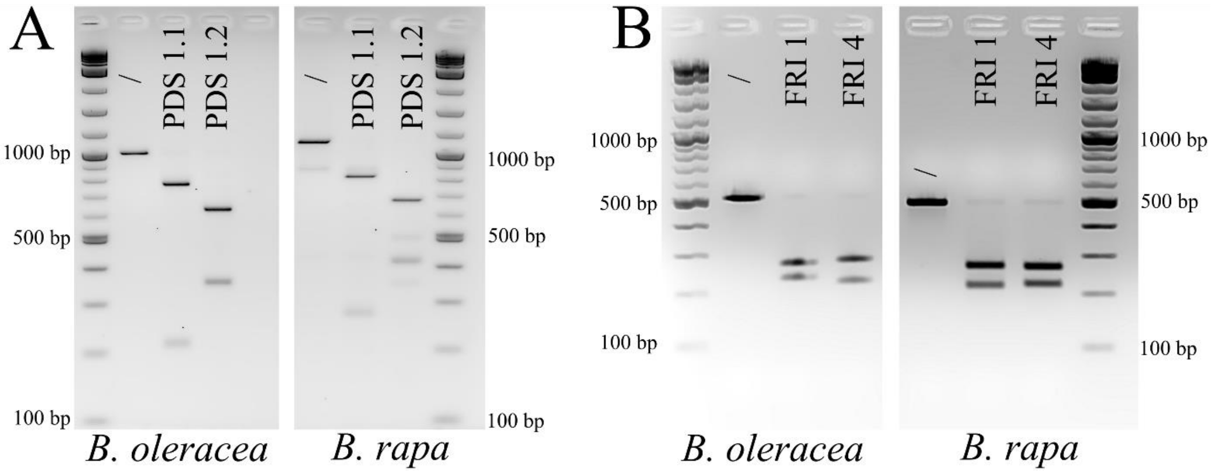

Supplementary Figure 2 Results of *in vitro* digestion assay of genes *PDS* (A) and *FRI* (B) amplified from cabbage (*B.oleracea*) and Chinese cabbage (*B. rapa*) DNA.

DNA-Free Genome Editing of *Brassica oleracea* and *Brassica rapa* Protoplasts Using CRISPR-Cas9 Ribonucleoprotein Complexes

Jana Murovec, Katja Guček, Borut Bohanec, Monika Avbelj, Roman Jerala

SUPPLEMENTARY MATERIAL

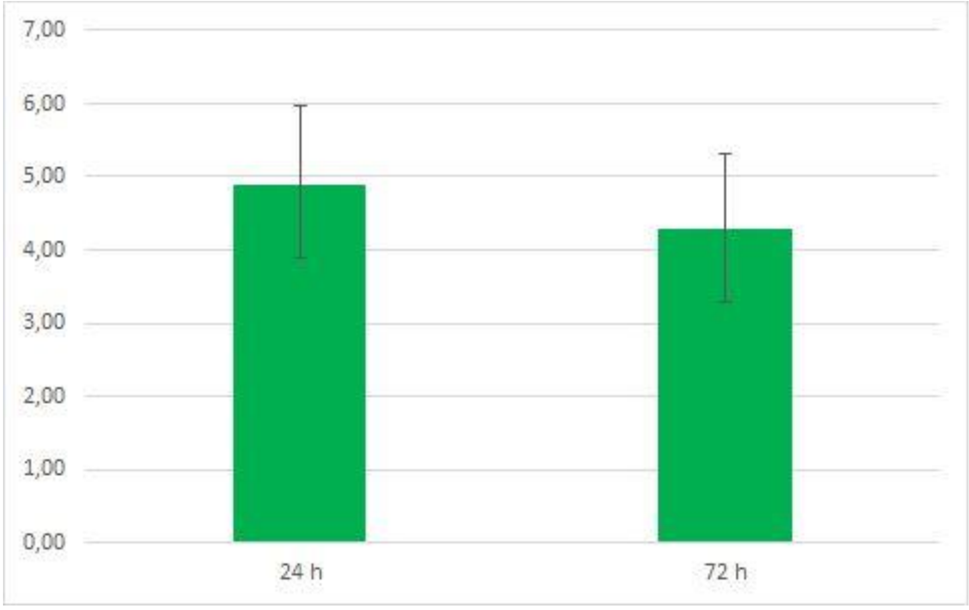

Supplementary Figure 3 Mutation frequencies in *B. rapa* protoplasts 24 hours or 72 hours after transfection of 15 µg of sgRNA-PDS2 and 15 µg of Cas9.
